# Exaggerated groups: amplification in ensemble coding of temporal and spatial features

**DOI:** 10.1098/rspb.2017.2770

**Published:** 2018-05-23

**Authors:** Shoko Kanaya, Masamichi J. Hayashi, David Whitney

**Affiliations:** 1 Department of Psychology, University of California, Berkeley, CA, USA; 2 Graduate School of Human and Environmental Studies, Kyoto University, Kyoto, Japan; 3 Global Center for Medical Engineering and Informatics, Osaka University, Osaka, Japan

**Keywords:** ensemble coding, summary statistics, perception, size, temporal frequency

## Abstract

The human visual system represents summary statistical information (e.g. average) along many visual dimensions efficiently. While studies have indicated that approximately the square root of the number of items in a set are effectively integrated through this ensemble coding, how those samples are determined is still unknown. Here, we report that salient items are preferentially weighted over the other less salient items, by demonstrating that the perceived means of spatial (i.e. size) and temporal (i.e. flickering temporal frequency (TF)) features of the group of items are positively biased as the number of items in the group increases. This illusory ‘amplification effect’ was not the product of decision bias but of perceptual bias. Moreover, our visual search experiments with similar stimuli suggested that this amplification effect was due to attraction of visual attention to the salient items (i.e. large or high TF items). These results support the idea that summary statistical information is extracted from sets with an implicit preferential weighting towards salient items. Our study suggests that this saliency-based weighting may reflect a more optimal and efficient integration strategy for the extraction of spatio-temporal statistical information from the environment, and may thus be a basic principle of ensemble coding.

## 1. Introduction

While we often believe that our visual system can encode rich and fully detailed information even in a brief glance, psychophysical studies have suggested that our capacity is constrained by several attentional and cognitive factors. Many researchers, however, recently suggested that this apparent contradiction may be reconciled by the discovery of ensemble coding—a mode of visual processing in which we can extract summary statistical information of a specific visual dimension (e.g. average size of circles) from a group of varied items [1,2]. Previous reports have shown that the ensemble coding works for a variety of visual dimensions ranging from low-level properties, such as size, orientation and spatial position, to high-level properties, such as faces, direction of bodily movement and liveliness [3–9].

Although some of the early research suggested that extraction of statistical summary information may involve processing of the entire set of items in a display with distributed attention [10,11], recent studies have suggested that it is unlikely that all the items presented are uniformly weighted in the integration [7,12–16]. A recent meta-analysis suggested that at least √*N* items are effectively integrated in the ensemble perception of the average, given a set size of *N* objects [17]. This could mean that some specific items are weighted more than other items in the ensemble estimation. This seems to hold across a range of different feature properties and object types. It is not clear, however, how those effective items are determined.

Psychophysical models that have been used to estimate the effective number of items to be integrated, such as the ideal observer model and the equivalent-noise simulation, assumed that the items are randomly sampled [13,14,16,18]. These models are important, but they may not be entirely realistic because weighting could be biased by various factors such as allocation of visual attention during the averaging task [19]. One alternative strategy might be ‘saliency-based’ weighting, where observers put more weight on items that are more visually salient than the others.^1^ This strategy might be more adaptive than weighting random items. The reason is that salient items with higher signal-to-noise ratio are reliable and easy to detect, therefore ensemble calculations heavily based on those items would be faster and more efficient.

To identify whether random weighting or saliency-based weighting is used to acquire ensemble information, here we systematically examined the bias in the perceived mean values as a function of set size. While previous studies have characterized ensemble perception across a variety of visual spatial dimensions, it is unclear if there are summary statistical representations of purely temporal information. We therefore tested not only spatial but also temporal visual dimensions (size and temporal frequency (TF) of flicker) by manipulating both dimensions simultaneously. This allowed us to determine whether the weighting mechanisms for ensemble perception of space and time are operated by the same principle.

**Figure 1.**
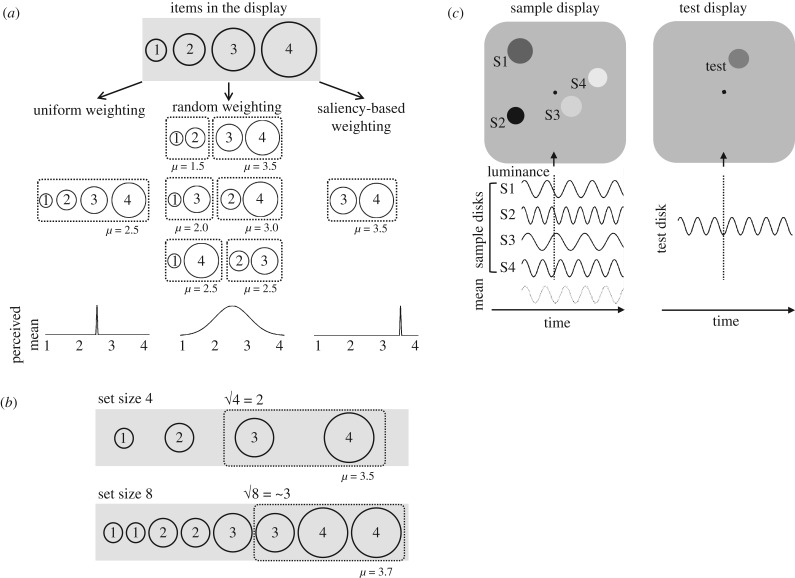
(*a*) Schematic predictions for the perceived mean values based on the three hypotheses. Dotted lines enclose a part of items (theoretically √*N* of the set size) weighted more than others. (*b*) An illustration of an increase in the perceived mean value according to the set size under the saliency-based weighting hypothesis. (*c*) Examples of the sample display and the subsequent test display in experiment 1 under one of the subset conditions (number of items presented = 4). Four sinusoidal waves in solid lines represent luminance modulations over time for the sample discs, and a dotted line at the bottom waves at the mean TF of them. The luminance of discs in the picture varies as they are in different phases of the sinusoidal waves (a vertical dotted line indicates the moment shown in the picture).

Figure 1*a* shows schematic predictions for the perceived mean values (i.e. in this example, mean size of items) based on the contrasting hypotheses (random weighting versus saliency-based weighting) in addition to another hypothetical strategy in which all items in the display are weighted uniformly (uniform weighting) as a baseline. Under the uniform weighting hypothesis, the perceived mean is expected to be equal to the mean of all items. Although the perceived mean for each trial fluctuates for random weighting, the centre of its distribution would be equal to the mean of all items. However, the saliency-based weighting strategy produces a positive bias in the perceived mean, because relatively salient (i.e. larger) items contribute more to the perception of the mean. Furthermore, an increase in the set size is expected to accentuate this positive bias (figure 1*b*). If √*N* salient items are weighted more than others, the proportion of items with relatively larger values in those prioritized members gradually increases according to the set size. Therefore, the perceived mean value should become larger and larger as the set size increases. Here, we refer to this as an amplification effect, a positive bias in the estimated mean due to ensemble coding of the sets.

## 2. General method

All participants were affiliates of the University of California, Berkeley, and gave informed consent to participate and received course credits or equivalent rewards as compensation for their time. Twenty observers participated in experiment 1. In experiment 2, 16 observers for the TF task and 15 observers for the size task participated. In experiment 3, 16 observers for the TF task and 16 independent participants for the size task participated. All participants had normal or corrected-to-normal vision. Data from one participant in the size task of experiment 1 and two participants in the size task of experiment 2 were excluded from group analyses. The reaction time data from one participant in the TF task and another participant in the size task of experiment 3 were excluded. See the electronic supplementary material for demographic information and exclusion criteria.

All the experiments were conducted in a quiet dark room at the University of California, Berkeley. The stimuli were presented using Psychophysics Toolbox v. 3 implemented in Matlab and run on iMac. Participants viewed stimuli on a CRT monitor (Samsung SyncMaster 997 DF) with resolution of 1024 × 768 and 60 Hz refresh rate. The viewing distance was kept constant at 57 cm using a chin-rest.

## 3. Experiment 1

Experiment 1 was designed to test two hypotheses simultaneously: (i) whether observers can integrate multiple TFs or sizes to estimate the mean flicker frequency or size of a crowd of flickering discs (ensemble coding hypothesis); and (ii) whether the estimated mean values show positive biases that increase as a function of the number of discs presented (amplification hypothesis).

### (a) Stimuli and procedure

Stimuli consisted of a variable number of discs presented on a grey background (figure 1*c*). The sample discs were distributed randomly across 14 possible locations—placeholders. The discs always flickered at various temporal frequencies regardless of the tasks.

All the participants performed two tasks: a TF discrimination task and a size discrimination task. Each task was performed in an independent session and the order of two tasks was counterbalanced between participants. In both tasks, each trial consisted of two intervals: the sample display and the test display. The sample display contained 1, 4, 8 or 14 discs (the 14 disc condition is the ‘whole set condition’). Sample discs were flickering at various temporal frequencies and had various sizes. The test display contained a single flickering disc at the center of the display. Durations of both sample and test displays were 3 s including ascending and descending luminance contrast ramps lasting 0.5 s each. Participants observed sample discs while fixating on a central fixation dot and compared the mean TF/size of the sample discs with the TF/size of the following test disc. They then reported which interval showed a higher TF/larger size by a key press. While both TFs and diameters of the sample and test discs varied, participants always knew which of those two dimensions they had to report, as they were informed at the beginning of each session which dimension is relevant to their task.

Flicker frequencies for discs were chosen from 20 TFs ranging from 0.5 to 12 Hz (electronic supplementary material, S1). They were spaced equally on a logarithmic scale. Sizes of discs were chosen from 20 diameters ranging from 1.5° to 3.37° visual angle, also spaced equally on a logarithmic scale. In each trial, a mean TF and a mean diameter were first randomly selected from 6 values ranging from the 13th to 8th highest of the 20 values. Next, 14 values that were −7, −6, −5, −4, −3, −2, −1, 1, 2, 3, 4, 5, 6 and 7 steps away from the mean were selected as the candidates for sample TFs/diameters. Among them, 1, 4, 8 or 14 TFs/diameters were selected randomly while no two discs in the sample display had the same TF/diameter.

The task-relevant parameter (i.e. flicker frequency in the TF task, diameter in the size task) for the test disc was one of 8 values that were −7, −5, −3, −1, 1, 3, 5 and 7 steps away from the mean with a constant probability (20 trials for each of eight test values per each set size condition), while the task-irrelevant parameter (i.e. diameter in the TF task, flicker frequency in the size task) for the test disc was matched to the mean of the sample values. Starting phases of the sinusoidal waves for sample flickers were randomized. The total number of trials for each task was 640 (4 set size conditions × 8 test values × 20 trials). For more details, see the electronic supplementary material.

### (b) Results

The proportion of trials where the test TFs/sizes were reported to be higher/larger than the mean was plotted as a function of the test steps relative to the mean of the whole set. The results from representative participants and fitted psychometric functions using a logistic equation are shown in the electronic supplementary material, S2. A psychometric function was fitted to each participant's data to obtain parameters for the slope and the point of subjective equality (PSE) that corresponds to the *x*-value that gives 50% of the proportion of ‘test higher/larger than the mean’ responses.

To test the ensemble coding hypothesis, we asked whether an observer's sensitivity in estimation of the mean TF/size improves when available information about that mean (i.e. set size) increases. The small subsets randomly chosen from 14 candidates in the subset conditions were not sufficient to fully represent the mean of the whole set (14 sample discs). Therefore, one's sensitivity against the mean of the whole set would be the lowest when the number of visible discs against full set is small (e.g. set size = 1) but gradually recovers as the number of visible discs increases (e.g. set size = 4, 8 or 14), if the participants can integrate multiple discs to compute the mean. However, if they fail to integrate multiple discs and randomly subsample only one of them, no matter how many discs are presented, the sensitivity against the mean would be consistently low and not vary as a function of the set size.

**Figure 2.**
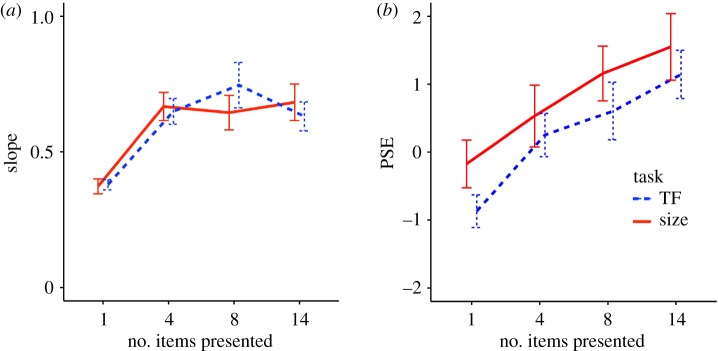
Averaged (*a*) slopes and (*b*) PSEs of the psychometric functions fitted to data of each individual participant in experiment 1. Error bars represent standard errors. (Online version in colour.)

A one-way repeated-measures ANOVA on the slopes of the psychometric functions (figure 2*a*) showed a significant main effect of the number of items presented for both of TF and size tasks (*F*_3,57_ = 12.62, *p* < 0.001, *η*^2^ = 0.24 for the TF task; *F*_3,54_ = 13.62, *p* < 0.001, *η*^2^ = 0.23 for the size task). A post-hoc test revealed that the slope parameters for the conditions with the 4, 8 and 14 items presented were significantly larger than that in the condition with only one disc presented for both tasks (*p* = 0.002 for the pair of the set size 1 and 4 conditions for the size task; *p* < 0.001 for the other comparisons). Other comparisons were not statistically significant. The steeper slopes for 4, 8 or 14 sets than that for a single disc suggest that integration of information in the multiple discs helped to compute the means in both tasks, supporting the notion of ensemble coding hypothesis. However, the fact that the slope stayed constant over 4, 8 and 14 sets indicates that the effective number of integrated discs was limited within the range of two to four discs.

Next, to test the amplification hypothesis, we focused on the reported values of mean TF or size. If relatively salient items (i.e. discs with large diameters or high TFs) contributed more to the perception of mean, the perceived means of a set of items would be larger than the TF/size of the averaged single items. Further, if the √*N* salient items are subsampled, the perceived mean value should become larger and larger as the set size increases.

A one-way repeated-measures ANOVA on perceived means of the presented items, reflected in PSEs (figure 2*b*), showed a significant main effect of the number of items presented for both of TF and size tasks (*F*_3,57_ = 15.83, *p* < 0.001, *η*^2^ = 0.20 for the TF task; *F*_3,54_ = 14.14, *p* < 0.001, *η*^2^ = 0.11 for the size task). For the TF task, a post-hoc test revealed significant differences between the PSE for a single disc and those for 4, 8 and 14 sets (*p* = 0.006 for set size 4; *p* = 0.012 for set size 8; *p* < 0.001 for set size 14) in addition to the difference between 4 sets and 14 sets (*p* < 0.001) and the difference between 8 sets and 14 sets (*p* = 0.037). As for the size task, the PSEs for the 8 sets and 14 sets were significantly higher than those for a single disc and 4 sets (*p* = 0.001 for set size 1 versus 4 and 2 versus 4; *p* = 0.002 for 1 versus 3; *p* = 0.007 for 2 versus 3). These results generally suggest that the perceived mean values for multiple discs were larger than the values reported for single discs, and this positive bias gradually increased as a function of the set size up to 14, supporting the amplification hypothesis.

## 4. Experiment 2

The results of experiment 1 were in line with the idea that observers integrated multiple objects, but weighted the relatively larger values more than the other items. However, one possible explanation for the positive bias of the estimated means is a response bias. If there is a common magnitude representation [23–25] for the relative size/speed/frequency of objects, any response bias in this dimension could manifest in ensemble judgements of both size and spatial frequency. Experiment 2 was designed to address this possibility by manipulating the variance of sample values in the two tasks for reporting averages (the TF task and the size task). If the amplification observed in experiment 1 was due to response bias, the gradual increase of the estimated mean size/TF according to the increase of the set size would be observed even when the variance in disc size/TF is zero (homogeneous sets). Conversely, if the amplification reflects the saliency-based weighting, the amplification of the perceived mean would be observed only in varied sets that include extremely salient items, not when the sets are composed of identical objects.

### (a) Stimuli and procedure

The stimuli and procedures were identical to those in experiment 1 except the following aspects. The order of the sample display and the test display was counterbalanced between participants in experiment 2. There were four conditions that included modulations in the variance of the sample values: the single condition, the homogeneous condition, the low-variance condition and the high-variance condition. A single disc was presented in the single condition while four discs were presented in the other three conditions. Sample discs flickered at various temporal frequencies and had various sizes regardless of the task. The positions of the sample discs and the test disc were randomly selected from the 14 placeholders. Half of the participants were instructed to report which interval showed a higher TF/larger size, while the other half were instructed to report an interval with a lower TF/smaller size by a key press.

In each trial, a mean TF/diameter was first randomly selected from 6 values using the same rule as in experiment 1. The mean TF and diameter were determined independently. Next, the sample TF(s)/diameter(s) were determined as follows. In the single condition, the mean TF/diameter itself was used for the single sample disc. In the homogeneous condition, the four sample TFs/diameters were all identical and equal to the mean TF/diameter itself. In the low-variance condition, the four sample TFs/diameters were four different values that were −3, −1, 1 and 3 steps away from the mean. In the high-variance condition, the four sample TFs/diameters were four different values that were −7, −5, 5 and 7 steps away from the mean. The test TF in the TF task or the test diameter in the size task was one of 8 values that were −7, −5, −3, −1, 1, 3, 5 and 7 steps away from the mean with a constant probability (16 trials for each of 8 test values per each set size condition), while the value of the other task-irrelevant dimension (e.g. the diameter in the TF task) for the test disc was matched to the mean of samples. Therefore, the total number of trials for each task was 512 (4 variance conditions × 8 test values × 16 trials).

### (b) Results

In experiment 2, we examined whether the amplification effect observed in experiment 1 is explained by a response bias or a cognitive correspondence of magnitude between mean values and set size. The cognitive correspondence hypothesis is that observers make judgements of larger or higher TF when there are more objects present; it is a form of response bias. We predicted that, if cognitive correspondence explains the amplification effect, the positive bias would be greater in homogeneous sets with no variance than the single sample discs while the bias would be comparable between high and low variance sets. By contrast, if the saliency-based weighting mediates the amplification effect, the positive biases would be enhanced in the high-variance sets compared with the low-variance sets while the positive bias would be equivalent between single and homogeneous sets.

The results from representative participants and fitted psychometric functions are shown in the electronic supplementary material, S3. As in experiment 1, a psychometric function was fitted to each participant's data to obtain the slope and the PSE.

**Figure 3.**
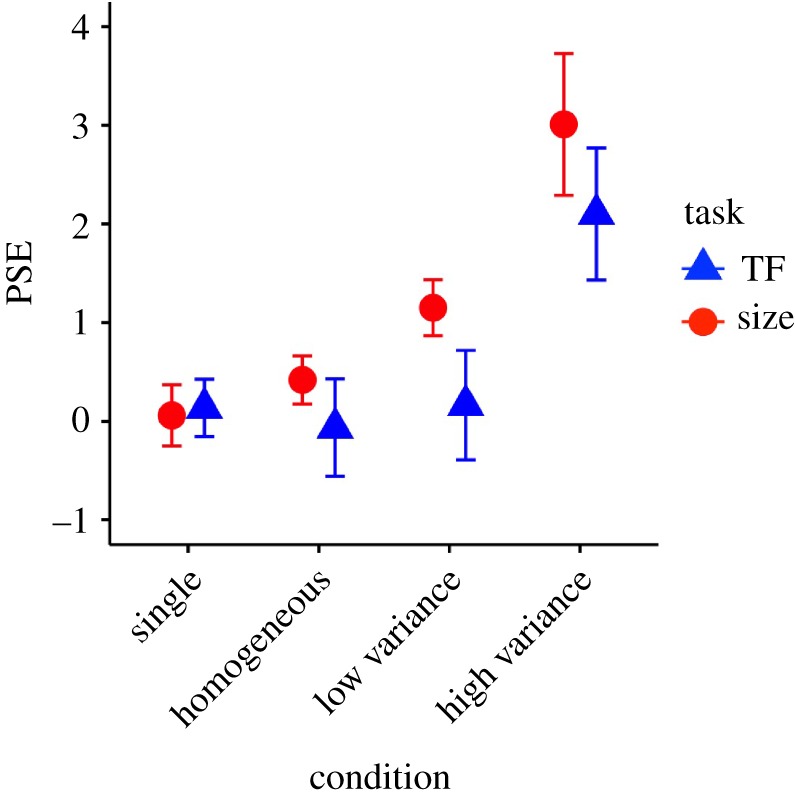
Averaged PSEs of the psychometric functions fitted to data of each individual participant in experiment 2. Error bars represent standard errors. (Online version in colour.)

A one-way repeated-measures ANOVA on the slope of the psychometric functions electronic supplementary material, S4 did not show any significant differences between the four conditions in the TF task or the size task. On the other hand, a significant main effect of the variance condition was found for the PSE (figure 3) both in the TF task (*F*_3,45_ = 15.00, *p* < 0.001, *η*^2^ = 0.16) and the size task (*F*_3,36_ = 13.16, *p* < 0.001, *η*^2^ = 0.36). A post-hoc test for the TF task revealed that the PSEs for the high-variance condition were significantly larger than those in the other three conditions (*p* = 0.006 for the pair of the single and high-variance condition; *p* < 0.001 for the other comparisons). As for the size task, the PSEs for the low-variance condition were significantly higher than those in the single and homogeneous conditions (*p* = 0.025 for both comparisons), and the PSEs for the high-variance condition were significantly higher than those in the other three conditions for the size task (*p* = 0.010 for the single and low variance conditions; *p* = 0.009 for the homogeneous condition).

In sum, this experiment provided two important findings. First, a simple increase of set size without increasing variance had no impact on perceived mean. This suggests that potential confounding of response bias, or a shared cognitive map between the set size and TF/diameter, is unlikely to explain our findings in experiment 1. Second, and most importantly, we found an enhanced amplification effect—positive bias in the perceived mean—when variance was increased, without changing the set size. This finding lends further support to the idea that amplification of perceived mean is mediated by preferential subsampling or weighting of relatively salient items.

## 5. Experiment 3

Experiments 1 and 2 demonstrated that the perceived mean TFs/sizes amplify as the set size increases only when samples are varied. This suggests that discs with larger values (TF or size) were preferentially weighted in the ensemble percept. However, one may argue that items with relatively small size/TF may be equally salient as items with relatively large size/high TF, given that the difference between the relevant item and its surroundings is the primary determinant of visual saliency [20]. Thus, the saliency-based weighting account may not explain the positive bias in the mean perception. To address this point, we examined the existence of asymmetry in the perceptual saliency between relatively large/high-TF items and small/low-TF items using a visual search paradigm. Participants looked for a target with a unique TF or diameter among homogeneous distractors and reaction time for detecting the target was evaluated. We compared three target TFs/diameters including low, middle and high values within the range used in experiments 1 and 2. We predicted that, if the discs with high TFs or large diameters were more salient and quickly attended than the remaining items, the search for the targets with relatively large values of TF/diameter would be faster and more efficient relative to the search for the targets with relatively low values.

### (a) Stimuli and procedure

Each trial consisted of a single search display following the presentation of a fixation dot for 0.5 s. The search display contained 4, 8 or 14 discs.

The discs in the TF search task flickered and were distributed on the 14 placeholders in the same way as in experiments 1 and 2. The flicker frequencies used in experiment 3 were 0.5, 2.45 and 12 Hz (low, middle and high TFs). In each trial, the single target disc flickered (random phase) at one of those three TFs (e.g. low TF). The other distractor discs flickered at one of the remaining two TFs (e.g. middle or high TF). The diameter of all the discs was fixed at 2.27° visual angle, which is equal to the centre of the range of diameters used in experiments 1 and 2. The duration of the search display in the TF task was 10 s including ascending and descending luminance contrast ramps lasting 0.5 s for each.

The stimuli in the size search task were static (non-flickering) white discs (38.88 cd m^−2^) on a 4 × 4 invisible matrix centred on the screen. The diameters of the discs were 1.88°, 2.27° and 2.74° in visual angle (small, middle and large diameters). Those diameters were spaced equally in a logarithmic scale in the range from the 16th to 5th largest value of those used in experiments 1 and 2. In each trial, the diameter of the single target disc was one of those three TFs (e.g. small diameter). The diameters of other distractor discs were one of the remaining two sizes (e.g. middle or large diameter). The duration of the search display in the TF task was 5 s including ascending and descending luminance contrast ramps lasting 0.5 s for each.

Participants observed the search display and clicked on the single target with a mouse cursor as quickly and accurately as possible. They were allowed to look around the whole display during the search, but were asked to fixate on the fixation dot until the search display appeared on each trial. The first position of the mouse cursor in each trial was always on the fixation dot. The search display was extinguished as soon as a participant clicked on the target or when the full duration of the search display had elapsed. Participants performed 20 trials for each combination so that the total number of trials was 360 (3 set sizes × 6 combinations × 20 trials). For more details, see the electronic supplementary material.

### (b) Results

Experiment 3 compared the visual search performances for three target TFs/diameters including low, middle and high values. If the discs with high TFs or large diameters were more salient and tended to pop-out more than the remaining items, the search for those items would be faster and more efficient relative to the search for the discs with relatively low TFs or small diameters.

Data from trials with a single target value were pooled across two different distractor TFs/diameters. A two-way repeated-measures ANOVA with the factors of target TF/diameter and set size on the proportion correct data (electronic supplementary material, S5) showed no main effects or interactions in either task, ensuring that there was no speed-accuracy trade-off between conditions. Therefore, the reaction time data from incorrect or miss (no-response) trials were excluded from further analysis.

**Figure 4.**
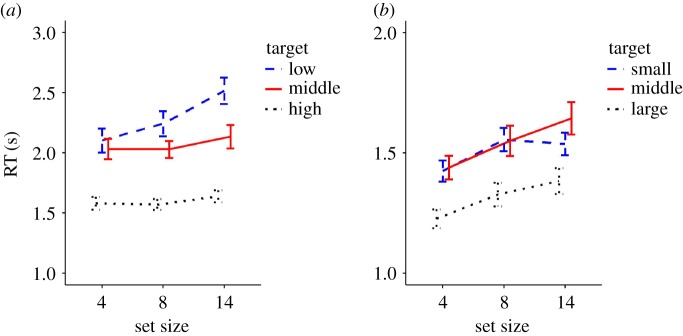
Averaged RTs for each target condition and set size in (*a*) the TF task and (*b*) the size task in experiment 3. Error bars represent standard errors. (Online version in colour.)

Figure 4*a* shows the RTs in the TF task as a function of the set size. A two-way repeated-measures ANOVA with the factors of target TF and set size showed a significant main effect of target TF (*F*_2,28_ = 63.91, *p* < 0.001, *η*^2^ = 0.44) and set size (*F*_2,28_ = 26.86, *p* < 0.001, *η*^2^ = 0.04) as well as a significant interaction (*F*_4,56_ = 6.24, *p* < 0.001, *η*^2^ = 0.02). A post-hoc test revealed that the simple main effect of set size was significant only for the low TF target. For the low TF target, the RTs for the three set sizes were significantly different from each other (*p* < 0.001 for 4 versus 14 and 8 versus 14; *p* = 0.019 for 4 versus 8), suggesting that the RT increased according to the set size. The simple main effects of the target TF were significant for all set sizes. For the set size 4, the high target TF showed a significantly shorter RTs than the other two target TFs (*p* < 0.001 for both comparisons) while there was no significant difference between low and middle TFs. The RTs for the three target TFs were significantly different from each other for the set size 8 (*p* < 0.001 for low versus high and middle versus high; *p* = 0.016 for low versus middle) and 14 (*p* < 0.001 for low versus high and middle versus high; *p* = 0.001 for low versus middle). To summarize, the target disc with the highest TF was localized with the shortest RTs regardless of the set size. The target with the middle TF was localized with the next shortest RTs regardless of the set size. The RTs for the low-TF target was comparable to that for the middle TF when the set size was 4, but increased according to the set size. The shorter RTs for the relatively high target TFs, as well as the independency of that effect on set size, suggests that the higher the target TF is, the more efficient the search for it was. The increasing RTs for the lowest target TF suggests that the search for extremely low TFs was relatively inefficient [20]. These results suggest that discs flickering at extremely high TFs were relatively more salient than the remaining discs on the display.

Figure 4*b* shows the RTs in the size search task as a function of the set size. A two-way repeated-measures ANOVA with the factors of target size and set size showed a significant main effect of target size (*F*_2,28_ = 35.35, *p* < 0.001, *η*^2^ = 0.20) and set size (*F*_2,28_ = 27.02, *p* < 0.001, *η*^2^ = 0.08), but no significant interaction. A post-hoc test revealed that the RTs for the large target diameter were significantly faster than the RTs for the other two diameters (*p* < 0.001 for both comparisons). Furthermore, the RTs for the three set sizes were significantly different from each other (*p* < 0.001 for all comparisons), suggesting that the RTs increased according to the set size. The target disc with the largest diameter was located faster than the targets with the other two diameters. The relatively faster detection of extremely large discs indicates that those larger discs were more salient than the remaining discs on the display.

## 6. Discussion

In the current study, we investigated which integration strategy, random weighting or saliency-based weighting, is used to represent the mean of a group of items, by measuring a bias in perceptual ensemble coding. In support of the saliency-based weighting hypothesis, we found a set-size-dependent increase in perceived mean that occurred both in spatial (i.e. size) and temporal (i.e. TF) dimensions (experiment 1). Our follow-up experiments ruled out the possibility that the amplification effect was a reflection of response bias by showing that a simple increase in set size with homogeneous sets resulted in no amplification effect (experiment 2); the amplification effect only occurs for sets with variability (in TF or size). Finally, visual search tasks with a similar set of stimuli as experiments 1 and 2 showed that search for targets with relatively high values of TF or size is generally faster and more efficient than search for targets with relatively low values. This corroborates the idea that large/high-TF items are preferentially weighted or subsampled over the other small/low-TF items. Taken together, these results provide strong evidence of saliency-based weighting mechanisms in spatial and temporal mean perception.

This is the first demonstration of amplification in summary statistical or ensemble perception in both spatial and temporal domains. Our findings may look counterintuitive, as some earlier papers reported that mean perception is fairly accurate regardless of the set size [4,26,27]. However, a number of previous studies are consistent with our attention-guided, saliency-based weighting account of the amplification effect. Several studies have reported that, although the ensemble coding can be achieved when attention cannot be fully deployed [26,28], attention still plays a substantial role in the ensemble coding [10,19,29]. For example, de Fockert & Marchant [19] reported that the size of a single attended item in the display systematically modulated the perceived mean size of the whole items [19]. Li & Yeh [30] found that the items on the left side of the display have larger impacts on the perceived ensemble properties than those on the other side of the display, as the left side is attentionally prioritized [30]. When considering that it is unlikely that all the items presented are weighted uniformly in the ensemble processing, one potential interpretation of their findings is that either exogenously or endogenously attended items are weighted more than the other items. Consistent with this view, the present study provides direct evidence for preferential weighting for salient items by showing that the effective number of items integrated was limited to around 4 or approximately √*N* of the maximum set size of 14, and, additionally, the perceived mean values were amplified according to the set size. The reason why the amplification effect has not been manifested in the previous literature could be that the perceived mean size was not directly measured in these studies [4,26,27]. Therefore, it is possible that the amplification was actually present in their data, although it was not obvious due to their experimental procedures and analyses.

The present study also revealed successful ensemble coding for visual TF for the first time. So far, while studies have revealed that a variety of spatial features can be integrated into ensemble representations even in temporally complex contexts such as for motion [7,31], surprisingly few studies have focused on ensemble processes for strictly temporal information. However, given that temporal features such as frequency and duration are the fundamental components of our visual perception, to investigate ensemble processes for those temporal features would lead to a deeper understanding of the efficient perception of the dynamic and continuous scenes surrounding us. Also, our findings are relevant for studies in the context of time perception. The effective number of flickering discs that were integrated—around two to four items presented in different spatial locations—might reflect the maximum number of temporal events that can be tracked simultaneously [32,33]. The ensemble representation of visual flicker may be critical for the parallel perception of multiple temporal signals.

We made it possible to compare ensemble perception of size and TF, to a certain extent, as the same stimuli were used for both tasks. Regardless of small differences between the results for TF and size, the general trends were similar for both of them throughout the three experiments. This suggests that the mechanisms for ensemble perception of TF and size have similar characteristics at least in terms of integration efficiency and weighting. Nevertheless, different mechanisms may underlie ensemble coding for different visual features [34,35]. We should note that our stimuli, which varied in TF and size at the same time, could have made it difficult for the participants to attend to one of those dimensions independently. Slightly different results, or different integration efficiency, might be observed under stimulus parameters that are optimized for the perception of each dimension *per se*.

The effective number of items integrated (2–4) is roughly in accordance with the idea that √*N* items are subsampled, as the maximum set size in experiment 1 was 14. Though the slopes in experiment 1 did not show an ideal exponential increase as a function of set size, this is probably because the range of the set size we used was not very large. Fig. 4 of Whitney & Yamanashi Leib [17] plotted the effective number of items as a function of set size for a variety of published studies, and found a best-fitted power function (*f*(*x*) = *x*^0.58^). This global trend was found by accumulating results from 20 published studies using a wide range of set size from a few to over 1000 at the maximum [16]. Therefore, the absence of a clear trend in a single empirical study does not contradict their claim.

One of the limitations revealed here is that very few studies of ensemble perception have used large set sizes, perhaps because of resolution and crowding constraints [36]. Developing new approaches could help. For example, an interesting recent technique we might call ensemble statistical learning found that observers can implicitly learn summary statistical information in larger set sizes [37,38]. Unlike ensemble statistical learning, the ensemble *perception* measured here and in most other studies occurs within a fraction of a second, within a single stimulus exposure, and manifests in appearance [1,2]. Nevertheless, combining new approaches like ensemble statistical learning with our ensemble perception approach could reveal how the visual system makes use of summary statistical information at many stages of processing, both explicitly (for appearance) as well as implicitly for learning and memory.

As the perceptual amplification effect we report here operates for two basic spatial and temporal visual dimensions, there is a possibility that the same principle applies for other low-level visual features. However, it is still unclear whether the amplification is observed for higher-level objects such as facial properties. Walker & Vul [39] suggested that the cheerleader effect, where faces in a group look more attractive than individuals, arises through the ensemble coding. Although the underlying mechanisms of the cheer-leader effect may be different from the amplification effect we report, the enhanced attractiveness of faces in a group could be relevant [40]. Testing higher-level stimuli (like faces) with our approach would be a useful future experiment.

Ensemble perception happens in many domains, across many stimuli, and may involve the integration of a proportion of the objects [5,7,12–16,18]. How the integration process itself operates has been unclear and debated [35,41,42]. Our results suggest that ensemble coding involves a weighted integration of objects, with relatively salient objects counting more towards the summary statistical representation. There are several reasons to think that this may be advantageous to the visual system. Preferentially integrating the stronger or more reliable signals would be faster and more efficient and it might provide a more flexible mechanism for representing summary statistical information at multiple levels of visual analysis. It does not undermine the usefulness of ensemble perception; on the contrary, weighted integration satisfies the competing needs of speed and efficiency, while also maximizing the representational veracity of the computed summary statistic. To get a gist of the crowd, one need not sample and integrate every single object; because of the benefit of averaging, a weighted sample of a proportion of the objects will provide an accurate ensemble representation of the scene.

## Supplementary Material

Supplementary methods, figures and tables

## Supplementary Material

Data Experiment 1

## Supplementary Material

Data Experiment 2

## Supplementary Material

Data Experiment 3

## Endnote

1 We use the term ‘saliency’ as a rather general concept that refers to how much an item attracts an observer's visual attention. Generally, basic features that sufficiently differ from their surroundings attract attention [20]. Furthermore, this effect is enhanced when the item has a larger value than its surroundings compared with when it has a smaller value than the surroundings, for some feature dimensions such as size and flicker frequency [21,22]. Those items expected to attract attention more than others are referred to as ‘salient’ items.

## References

[1] Whitney D, Haberman J, Sweeny T 2014 From textures to crowds: multiple levels of summary statistical perception. In The new visual neuroscience (eds Werner JS, Chalupa LM), pp. 695–710. Cambridge, MA: MIT Press.

[2] Alvarez GA 2011 Representing multiple objects as an ensemble enhances visual cognition. Trends Cogn. Sci. 15, 122–131. (10.1016/j.tics.2011.01.003) 21292539

[3] Chong SC, Treisman A 2003 Representation of statistical properties. Vis. Res. 43, 393–404. (10.1016/S0042-6989(02)00596-5) 12535996

[4] Ariely D 2001 Seeing sets: representation by statistical properties. Psychol. Sci. 12, 157–162. (10.1111/1467-9280.00327) 11340926

[5] Leib AY, Kosovicheva A, Whitney D 2016 Fast ensemble representations for abstract visual impressions. Nat. Commun. 7, 13186 (10.1038/ncomms13186) 27848949 PMC5116093

[6] Haberman J, Whitney D 2009 Seeing the mean: ensemble coding for sets of faces. J. Exp. Psychol. Hum. Percept. Perform. 35, 718–734. (10.1037/a0013899) 19485687 PMC2696629

[7] Sweeny TD, Haroz S, Whitney D 2013 Perceiving group behavior: sensitive ensemble coding mechanisms for biological motion of human crowds. J. Exp. Psychol. Hum. Percept. Perform. 39, 329–337. (10.1037/a0028712) 22708744

[8] Parkes L, Lund J, Angelucci A, Solomon JA, Morgan M 2001 Compulsory averaging of crowded orientation signals in human vision. Nat. Neurosci. 4, 739–744. (10.1038/89532) 11426231

[9] Morgan MJ, Glennerster A 1991 Efficiency of locating centres of dot-clusters by human observers. Vis. Res. 31, 2075–2083. (10.1016/0042-6989(91)90165-2) 1771793

[10] Chong SC, Treisman A 2005 Attentional spread in the statistical processing of visual displays. Atten. Percept. Psychophys. 67, 1–13. (10.3758/BF03195009) 15912869

[11] Chong SC, Joo SJ, Emmanouil TA, Treisman A 2008 Statistical processing: not so implausible after all. Percept. Psychophys. 70, 1327–1334; discussion 1335–1326 (10.3758/PP.70.7.1327) 18927015

[12] Allik J, Toom M, Raidvee A, Averin K, Kreegipuu K 2013 An almost general theory of mean size perception. Vis. Res. 83, 25–39. (10.1016/j.visres.2013.02.018) 23499976

[13] Maule J, Franklin A 2016 Accurate rapid averaging of multihue ensembles is due to a limited capacity subsampling mechanism. J. Opt. Soc. Am. A Opt. Image Sci. Vis. 33, A22–A29. (10.1364/JOSAA.33.000A22) 26974927

[14] Im HY, Halberda J 2013 The effects of sampling and internal noise on the representation of ensemble average size. Atten. Percept. Psychophys. 75, 278–286. (10.3758/s13414-012-0399-4) 23188732

[15] Gorea A, Belkoura S, Solomon JA 2014 Summary statistics for size over space and time. J. Vis. 14, 22 (10.1167/14.9.22) 25157045

[16] Dakin SC 2001 Information limit on the spatial integration of local orientation signals. J. Opt. Soc. Am. A 18, 1016–1026. (10.1364/JOSAA.18.001016) 11336204

[17] Whitney D, Yamanashi Leib A 2018 Ensemble perception. Annu. Rev. Psychol. 69, 1–25. (10.1146/annurev-psych-010416-044232) 28892638

[18] Florey J, Clifford CW, Dakin S, Mareschal I 2016 Spatial limitations in averaging social cues. Sci. Rep. 6, 32210 (10.1038/srep32210) 27573589 PMC5004154

[19] de Fockert JW, Marchant AP 2008 Attention modulates set representation by statistical properties. Percept. Psychophys. 70, 789–794. (10.3758/PP.70.5.789) 18613627

[20] Wolfe JM, Horowitz TS 2017 Five factors that guide attention in visual search. Nat. Hum. Behav. 1, 0058 (10.1038/s41562-017-0058) PMC9879335 36711068

[21] Ivry RB, Cohen A 1992 Asymmetry in visual search for targets defined by differences in movement speed. J. Exp. Psychol. Hum. Percept. Perform. 18, 1045–1057. (10.1037/0096-1523.18.4.1045) 1431743

[22] Proulx MJ 2010 Size matters: large objects capture attention in visual search. PLoS ONE 5, e15293 (10.1371/journal.pone.0015293) 21203454 PMC3009719

[23] Price PC, Kimura NM, Smith AR, Marshall LD 2014 Sample size bias in judgments of perceptual averages. J. Exp. Psychol. Learn. Mem. Cogn. 40, 1321–1331. (10.1037/a0036576) 24749965

[24] Smith AR, Price PC 2010 Sample size bias in the estimation of means. Psychon. Bull. Rev. 17, 499–503. (10.3758/PBR.17.4.499) 20702868

[25] Walsh V 2003 A theory of magnitude: common cortical metrics of time, space and quantity. Trends Cogn. Sci. 7, 483–488. (10.1016/j.tics.2003.09.002) 14585444

[26] Chong SC, Treisman A 2005 Statistical processing: computing the average size in perceptual groups. Vis. Res. 45, 891–900. (10.1016/j.visres.2004.10.004) 15644229

[27] Robitaille N, Harris IM 2011 When more is less: extraction of summary statistics benefits from larger sets. J. Vis. 11, 18 (10.1167/11.12.18) 22031908

[28] Alvarez GA, Oliva A 2008 The representation of simple ensemble visual features outside the focus of attention. Psychol. Sci. 19, 392–398. (10.1111/j.1467-9280.2008.02098.x) 18399893 PMC2587223

[29] Dakin SC, Bex PJ, Cass JR, Watt RJ 2009 Dissociable effects of attention and crowding on orientation averaging. J. Vis. 9, 1–16. (10.1167/9.11.28) PMC2927104 20053091

[30] Li KA, Yeh SL 2017 Mean size estimation yields left-side bias: role of attention on perceptual averaging. Atten. Percept. Psychophys. 79, 2538–2551. (10.3758/s13414-017-1409-3) 28842864

[31] Albrecht AR, Scholl BJ 2010 Perceptually averaging in a continuous visual world: extracting statistical summary representations over time. Psychol. Sci. 21, 560–567. (10.1177/0956797610363543) 20424102

[32] Cheng X, Yang Q, Han Y, Ding X, Fan Z 2014 Capacity limit of simultaneous temporal processing: how many concurrent ‘clocks’ in vision? PLoS ONE 9, e91797 (10.1371/journal.pone.0091797) 24632675 PMC3954791

[33] Holcombe AO 2009 Seeing slow and seeing fast: two limits on perception. Trends Cogn. Sci. 13, 216–221. (10.1016/j.tics.2009.02.005) 19386535

[34] Haberman J, Brady TF, Alvarez GA 2015 Individual differences in ensemble perception reveal multiple, independent levels of ensemble representation. J. Exp. Psychol. Gen. 144, 432–446. (10.1037/xge0000053) 25844624

[35] Hubert-Wallander B, Boynton GM 2015 Not all summary statistics are made equal: evidence from extracting summaries across time. J. Vis. 15, 5 (10.1167/15.4.5) PMC4463776 26053144

[36] Whitney D, Levi DM 2011 Visual crowding: a fundamental limit on conscious perception and object recognition. Trends Cogn. Sci. 15, 160–168. (10.1016/j.tics.2011.02.005) 21420894 PMC3070834

[37] Chetverikov A, Campana G, Kristjansson A 2016 Building ensemble representations: how the shape of preceding distractor distributions affects visual search. Cognition 153, 196–210. (10.1016/j.cognition.2016.04.018) 27232163

[38] Chetverikov A, Campana G, Kristjansson A 2017 Set size manipulations reveal the boundary conditions of perceptual ensemble learning. Vis. Res. 140, 144–156. (10.1016/j.visres.2017.08.003) 28987926

[39] Walker D, Vul E 2014 Hierarchical encoding makes individuals in a group seem more attractive. Psychol. Sci. 25, 230–235. (10.1177/0956797613497969) 24163333

[40] Post RB, Haberman J, Iwaki L, Whitney D 2012 The frozen face effect: why static photographs may not do you justice. Front. Psychol. 3, 22 (10.3389/fpsyg.2012.00022) 22363302 PMC3282501

[41] Haberman J, Whitney D 2010 The visual system discounts emotional deviants when extracting average expression. Atten. Percept. Psychophys. 72, 1825–1838. (10.3758/APP.72.7.1825) 20952781 PMC3123539

[42] De Gardelle V, Summerfield C 2011 Robust averaging during perceptual judgment. Proc. Natl Acad. Sci. USA 108, 13 341–13 346. (10.1073/pnas.1104517108) PMC3156162 21788517

