# Supplementary material for: Exaggerated groups: amplification in ensemble coding of temporal and spatial features
Source: Proc Biol Sci. 2018 May 23;285(1879):20172770. doi: 10.1098/rspb.2017.2770 (PMC5998104; doi:10.1098/rspb.2017.2770)
Supplement: Supplementary methods, figures and tables [file rspb20172770supp1.docx]

**Exaggerated groups: Amplification in ensemble coding of temporal and spatial features**

Shoko Kanaya^1,2^, Masamichi J Hayashi^1,3^, David Whitney^1^

^1^University of California Berkeley, ^2^Kyoto University, ^3^Osaka University

*Corresponding Author: shoko.kanaya@berkeley.edu

**Supplementary Methods**

**Supplementary Figures and Tables**

**Experiment 1**

**Participants**

Participants were 8 males and 12 females including one of authors, SK (mean age = 23.70, SD = 6.23). All but this author were naive regarding the purpose of the experiment.

**Stimuli**

The luminance of the gray background was 23.86 cd/m^2^. The placeholders were virtual disks arranged on two concentric rings with radii of 4° and 8°visual angle, measured from a fixation dot at the center of the display. The inner ring contained 6 placeholders and the outer ring contained 8 placeholders that were equidistantly spaced. The stimulus disks were placed in some or all of those placeholders with random jitter. The maximum size of jitter varied from 0°(for the largest disk) to 1.84°(for the smallest disk), and different disks in the same display never overlapped. The luminance of disks was changed sinusoidally within the range of 14.03 – 38.88 cd/m^2^.

**Data analysis**

Data from one participant suggested that he reported the test size larger than the mean in more than 50% of trials for all the test values in some conditions. Therefore, data from this participant was excluded from the group analyses for the size task.

**Experiment 2**

**Participants**

Participants who performed the TF task were 5 males and 11 females including one of authors, SK (mean age = 20.81, SD = 3.15). Participants who performed the size task were 4 males and 11 females including one of authors, SK (mean age = 21.2, SD = 3.14). This author performed both tasks while others performed only one of the tasks. All but this author were naive regarding the purpose of the experiment.

**Data analysis**

Data from two participants in the size task were excluded from the group analysis, as these subjects reported the test systematically smaller than the mean in every condition.

**Experiment 3**

**Participants**

Participants who performed the TF search task were 10 males and 6 females (mean age = 21.06, SD = 2.29). Participants who performed the size search task were 7 males and 9 females (mean age = 19.56, SD = 5.69). All of them were naive regarding the purpose of the experiment.

**Stimuli**

The matrix on which the stimuli for the size search task were presented was 16° x 16°wide, and disks were placed in some of the cells with a random jitter. The maximum sizes of the jitter were 1.92°, 1.53°, and 1.06° for the small, middle, and large disks respectively. The 14 placeholders used in the TF search task were not used in this task because a target which is larger or smaller than the distractor disks would change the global configuration of placeholders arranged on two concentric rings and affect search performance in an unpredictable manner. The range of diameters used in the size search task was narrower than that in Experiment 1 and 2 to make the search task adequately difficult.

**Data analysis**

The reaction time data from one participant in the TF task and another participant in the size task were excluded, as they showed RTs longer than 3 SDs of the mean in some conditions.

S1. Twenty steps of TFs and sizes that were used in Experiment 1. The mean TF or diameter in each trial was randomly selected from the 6 steps ranging from the 13^th^ to 8^th^ highest of them. The TFs or diameters for sample disks (1, 4, 8, or 14) were chosen from 14 steps ranging from -7 to +7 steps away from the mean but not including the mean itself.


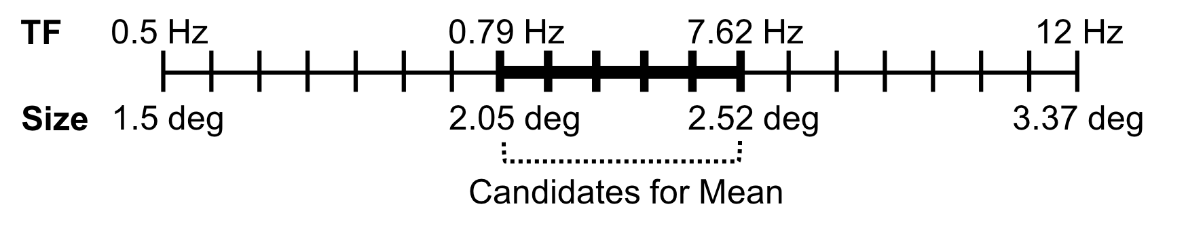


S2. Proportion of trials where the test TF/size higher/larger than the mean of sample disks was reported in Experiment 1. Data from two representative participants are shown for the TF and size tasks respectively. Solid lines represent fitted psychometric functions.

S3. Proportion of trials where the test TF/size was reported as higher/larger than the mean of sample disks in Experiment 2. Data from two representative participants are shown for the TF and size tasks respectively. Solid lines represent fitted psychometric functions.

S4. Averaged slopes of the psychometric functions fitted to data of each individual participant in Experiment 2. Error bars represent standard errors.


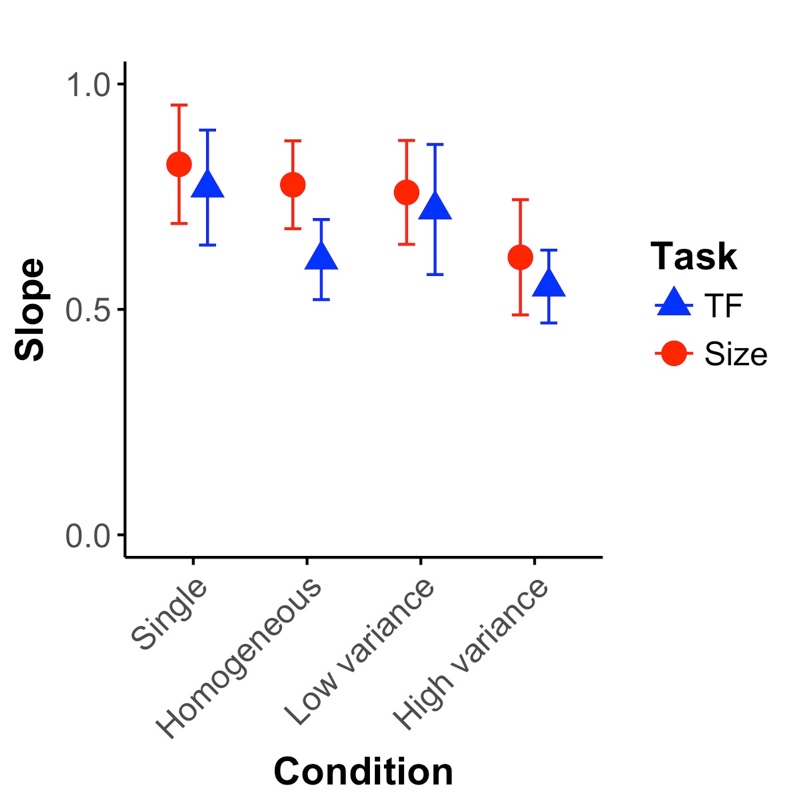


S5. The averaged proportion correct for each target condition and set size in the visual search tasks in Experiment 3. Standard deviations are in the parentheses.

|  | TF task | | | Size task | | |
| --- | --- | --- | --- | --- | --- | --- |
|  | Target | | | Target | | |
| Set Size | Low | Middle | High | Small | Middle | Large |
| 4 | 0.95(0.05) | 0.94(0.05) | 0.97(0.03) | 0.96(0.04) | 0.97(0.03) | 0.97(0.03) |
| 8 | 0.95(0.05) | 0.94(0.05) | 0.97(0.04) | 0.97(0.02) | 0.95(0.03) | 0.98(0.03) |
| 14 | 0.94(0.06) | 0.94(0.05) | 0.97(0.03) | 0.97(0.03) | 0.98(0.02) | 0.97(0.03) |
